# Comparison of different colistin sulfate regimens for carbapenem-resistant gram-negative bacteria pneumonia in neurocritical care patients: a retrospective cohort study

**DOI:** 10.1128/aac.00644-25

**Published:** 2025-09-22

**Authors:** Qian Zeng, Huawei Huang, Jiaqi Lu, Lei Wu, Shaolan Zhang, Jingwei Zhao, Guangqiang Chen, Hongliang Li, Guangzhi Shi

**Affiliations:** 1 Department of Intensive Care Unit, Beijing Tiantan Hospital, Capital Medical University 105738 , Beijing, China; 2 Department of Intensive Care Unit, Beijing Shijitan Hospital, Capital Medical University 117968, Beijing, China; 3 Emergency and Critical Care Medical Center, Beijing Shijitan Hospital, Capital Medical University 12517, Beijing, China; University of Fribourg, Fribourg, Switzerland

**Keywords:** nebulized, colistin sulfate, nosocomial pneumonia, carbapenem-resistant gram-negative bacteria, neurocritical care

## Abstract

Nosocomial infection caused by carbapenem-resistant gram-negative bacteria (CR-GNB) is a common problem in neurocritical care patients. Clinical evidence suggests that polymyxins have benefits for CR-GNB pneumonia. This study compared the efficacy and safety of different colistin sulfate regimens in CR-GNB pneumonia. Among 133 neurocritical care patients with CR-GNB pneumonia in the intensive care unit (ICU), 24 received nebulized colistin sulfate alone (NC group); 38 received intravenous colistin sulfate alone (IV group); and 71 received nebulized plus intravenous colistin sulfate (NCIV group). After inverse probability of treatment weighting (IPTW), clinical failure rates on days 7 and 14 were significantly higher in the IV group than in the NC group (38.3% vs. 20.5%, *P* = 0.017 and 32.1% vs. 15.3%, *P* = 0.004, respectively) and the NCIV group (38.3% vs. 24.7%, *P* = 0.023 and 32.1% vs. 14.2%, *P* = 0.015, respectively). Moreover, the IV group also reported a lower microbiological eradication rate on day 14 (*P* = 0.031) and longer ICU (*P* = 0.020) and hospital stays (*P* = 0.037) than the NCIV group. No significant difference in mortality risk and nephrotoxicity among the groups. In multivariable analysis, intravenous colistin sulfate was an independent factor associated with higher clinical failure on day 14 (adjusted odds ratio = 5.92, 95% CI = 1.14–30.84, *P* = 0.035). Our study suggested that nebulized colistin sulfate with concurrent intravenous administration may be an effective and safe option for CR-GNB pneumonia.

## INTRODUCTION

Hospital-acquired pneumonia (HAP) and ventilator-associated pneumonia (VAP) are the main nosocomial infections with high rates of morbidity and mortality (1). Owing to the severity of HAP and VAP in the intensive care unit (ICU), early appropriate antimicrobial therapy significantly improves the survival of patients (2). In recent years, the overuse and misuse of broad-spectrum antibiotics have accelerated the spread of antibiotic-resistant bacteria. The dramatic increase in multidrug-resistant (MDR) bacteria represented by gram-negative bacteria (GNB) has complicated the management, therapy, and outcome of nosocomial infections. Among these pathogens, carbapenem-resistant GNB (CR-GNB), especially CR-*Acinetobacter baumannii* (CRAB), CR-*Enterobacteriaceae* (CRE), and CR-*Pseudomonas aeruginosa* (CRPA), have gained attention with growing public health challenges worldwide (1).

Polymyxins, primarily polymyxin B (PMB) and polymyxin E, are old antibiotics discovered in 1947 that are effective against MDR-GNB (3). Polymyxin E, commonly known as colistin, has been available for clinical use as colistin methanesulfonate sodium (CMS) and colistin sulfate. CMS is an essentially inactive prodrug hydrolyzed to produce colistin, whereas colistin sulfate is only used for intravenous administration in China (3). However, dose-dependent nephrotoxicity and neurotoxicity are the most common adverse events of intravenous polymyxins, which limit their widespread clinical application (4).

Studies have reported low penetration of intravenous colistin in lung tissue, resulting in inadequate antibacterial activity (5, 6). Nebulized colistin has been used in patients diagnosed with cystic fibrosis for decades and has obtained satisfactory clinical effects (7). Lu et al. (8) found a significantly higher concentration in the lung tissue after nebulization than after intravenous administration of colistin. Additionally, nebulized colistin can significantly reduce systemic exposure and provide the possibility to increase doses without increasing the risk of toxicity (6). In 2018, a meta-analysis demonstrated the effectiveness and safety of inhaled colistin as monotherapy for treating respiratory tract infections caused by MDR or colistin-only susceptible GNB (9). Nevertheless, the lack of case-control studies limits these findings. Different studies have compared the efficacy of intravenous therapy alone versus intravenous therapy combined with nebulized CMS, with varying results. Two meta-analyses showed that nebulized CMS as adjunctive therapy had additional benefits compared with intravenous CMS alone (10, 11). However, a further meta-analysis by Vardakas et al. (12) did not confirm these benefits. Currently, most studies have focused on the administration of CMS, but few studies have investigated the clinical efficacy and safety of colistin sulfate.

Pneumonia is a common complication among neurocritical care patients and may be caused by their consciousness impairment, prolonged bed rest, and immunosuppression due to brain injury. The role of neurologic disease as an independent risk factor for the development of VAP has been previously established (13). A retrospective study revealed that 44.9% of mechanically ventilated patients in the neurosurgical ICU developed VAP within 7 days (14). Therefore, this study aimed to assess the associations between colistin sulfate and treatment outcomes in neurocritical care patients with nosocomial pneumonia caused by CR-GNB.

## MATERIALS AND METHODS

### Study design and patients

This retrospective cohort study was conducted at the ICU of Beijing Tiantan Hospital from June 2020 to April 2024. ICU-admitted patients with pneumonia caused by CR-GNB were eligible for enrollment. Furthermore, neurocritical care patients were defined as those with brain neoplasms, intracranial hemorrhage (ICH), ischemic stroke, subarachnoid hemorrhage (SAH), traumatic brain injury (TBI), spinal cord injury, or those undergoing neurosurgical procedures, such as craniotomy or spinal surgery. Based on the treatment regimens of colistin sulfate, patients were divided into three groups: the NC group (nebulized colistin sulfate alone), the IV group (intravenous colistin sulfate alone), and the NCIV group (nebulized combined with intravenous colistin sulfate). The inclusion criteria included: (i) neurocritical care patients diagnosed with pneumonia developing more than 48 h after admission; (ii) positive cultures of CR-GNB isolated from sputum or bronchoalveolar lavage fluid (BALF); and (iii) nebulized or intravenous administration of colistin sulfate for ≥3 days and initiated from the five preceding and following days after the date of the index culture study. The exclusion criteria were as follows: (i) age < 18 years; (ii) diagnosed with community-acquired pneumonia or concomitant lung cancer with obstructive pneumonitis; (iii) pregnancy, perinatal period, or feeding period; (iv) acute or chronic renal insufficiency at baseline; and (v) colistin-resistant CR-GNB.

The study protocol was approved by the Institutional Review Board of Beijing Tiantan Hospital Affiliated with Capital Medical University (approval number KY2022-246-02), which waived the requirement for written informed consent given the retrospective nature of the study and the use of anonymized data from hospital records. Moreover, the study followed the ethical standards of the institutional committee on human experimentation and the Helsinki Declaration of 1975.

### Diagnosis of pneumonia and microbiological tests

The diagnosis of pneumonia was based on a new lung infiltrate on chest imaging with clinical evidence that the infiltrate is of an infectious origin, which includes fever, purulent sputum, leukocytosis, and respiratory decline (1). HAP was defined as a new pneumonia occurring more than 48 h after admission in non-intubated patients, and VAP was defined as a new pneumonia developing after 48 h of endotracheal intubation and mechanical ventilation (1). Sputum or BALF samples were collected from each patient for bacterial culture or metagenomic next-generation sequencing (mNGS). Antimicrobial susceptibility testing was used to determine the minimum inhibitory concentration (MIC) according to previously published international guidelines (15). The isolate was considered a CR-GNB if phenotypically resistant to ≥1 carbapenem (16). The collection date of the index culture study was defined as the pneumonia index date.

### Data collection

Demographic characteristics were retrieved from electronic medical records. Other data collected included underlying comorbidities, isolated pathogenic bacteria, concomitant antibiotic therapy, the Glasgow Coma Scale (GCS) score, laboratory results, and the presence of organ dysfunction on the pneumonia index date, including septic shock and invasive ventilator. The collected laboratory results were as follows: leukocytes, neutrophil percentage, C-reactive protein (CRP), procalcitonin (PCT), albumin, serum creatinine, and glomerular filtration rate (GFR). Augmented renal clearance (ARC) was objectively defined as a creatinine clearance of more than 130 mL/min/1.73 m^2^ (17).

### Colistin sulfate regimens

In this study, all patients were treated with nebulized or intravenous colistin sulfate (Shanghai New Asia Pharmaceutical Co., Ltd., China). Bronchodilators were inhaled 30 min before colistin sulfate inhalation, which minimized the risk of bronchospasm and improved drug permeability. The daily dose of nebulized colistin sulfate was 250,000 international units every 12 h, and each dose was diluted in 10 mL of normal saline. It was administered immediately upon reconstitution for 20 min via a PARI nebulizer (PARI GmbH, Germany). In mechanically ventilated patients, the nebulizer was placed at 15 cm upstream of the Y-piece and connected to the inspiratory outlet of the ventilator. During the nebulization period, volume-controlled mode was suggested, and the conventional humidifier was off. Non-ventilated patients inhaled aerosol through a face mask connected to a nebulizer. Intravenous colistin sulfate of 500,000 international units was administered every eight or 12 h.

### Efficacy and safety evaluations

The efficacy evaluations included the primary and secondary outcomes. In this study, the primary endpoints were clinical responses and microbiological eradication rates on days 7, 14, and 28. Clinical responses were classified as cure (resolution of symptoms/signs of pneumonia, improvement or progression-free of chest radiographic abnormalities, and free from antibiotics), improvement (partial resolution of symptoms/signs of pneumonia, improvement or progression-free of chest radiographic abnormalities but not free from antibiotics), and failure (persistent or progression of symptoms/signs of pneumonia, persistent or worsening of radiographic abnormalities that required additional antibiotic therapy, or death) (18). The secondary endpoints included all-cause mortality on days 14 and 28, ventilator weaning rate on day 28, and days of ICU and hospital stay.

The safety outcome was based on renal function between the start and end times of treatment. The Kidney Disease: Improving Global Outcomes (KDIGO) criteria, which were assessed by the serum creatinine and urine output, were intended for use in the evaluation of nephrotoxicity (19).

### Inverse probability of treatment weighting (IPTW)

To adjust for confounding variables, we used IPTW, which has already been proven as an effective method in clinical retrospective studies (20–22), to deal with imbalances in the baseline characteristics of the treatment groups. Before IPTW, we performed the expectation-maximization (EM) algorithm to impute missing data. The propensity scores were calculated using a multivariable logistic regression model with all baseline covariates, and we then used the propensity score to reweight the cohort by stabilized IPTW. Finally, the standardized mean difference (SMD) was used to evaluate the weighting effect, and an SMD value ≤ 20% was considered acceptable. IPTW was fulfilled by R version 4.4.1 (R Foundation for Statistical Computing, Vienna, Austria).

### Statistical analysis

All analyses were performed using R. Demographic and clinical features were summarized with descriptive statistics. The Kolmogorov-Smirnov test was adopted to test for a normal distribution. Depending on the distributional properties of the data, the one-way analysis of variance (ANOVA) or Kruskal-Wallis test was used for comparisons among three groups, whereas the differences between two groups were evaluated by Student’s *t* test or Mann-Whitney *U* test. Categorical variables were compared using the *χ* or Fisher’s exact test.

After IPTW, the efficacy and safety outcomes for the three therapy groups were then examined using the weights. Generalized linear models were employed for response variables, and the associations of treatment with all‐cause mortality and the ventilator weaning rate were analyzed via the Cox proportional hazards regression. In addition, weighted Kaplan-Meier curves with log-rank tests were conducted to compare all‐cause mortality among the groups. Binary forward stepwise logistic regression was performed to determine the independent variables associated with day 14 clinical failure. Variables with a *P*-value < 0.1 in the univariable analysis were included in the subsequent multivariable model. All tests were two-tailed, and *P*-values < 0.05 were considered statistically significant.

## RESULTS

### Demographic characteristics and clinical features

**Fig 1:**
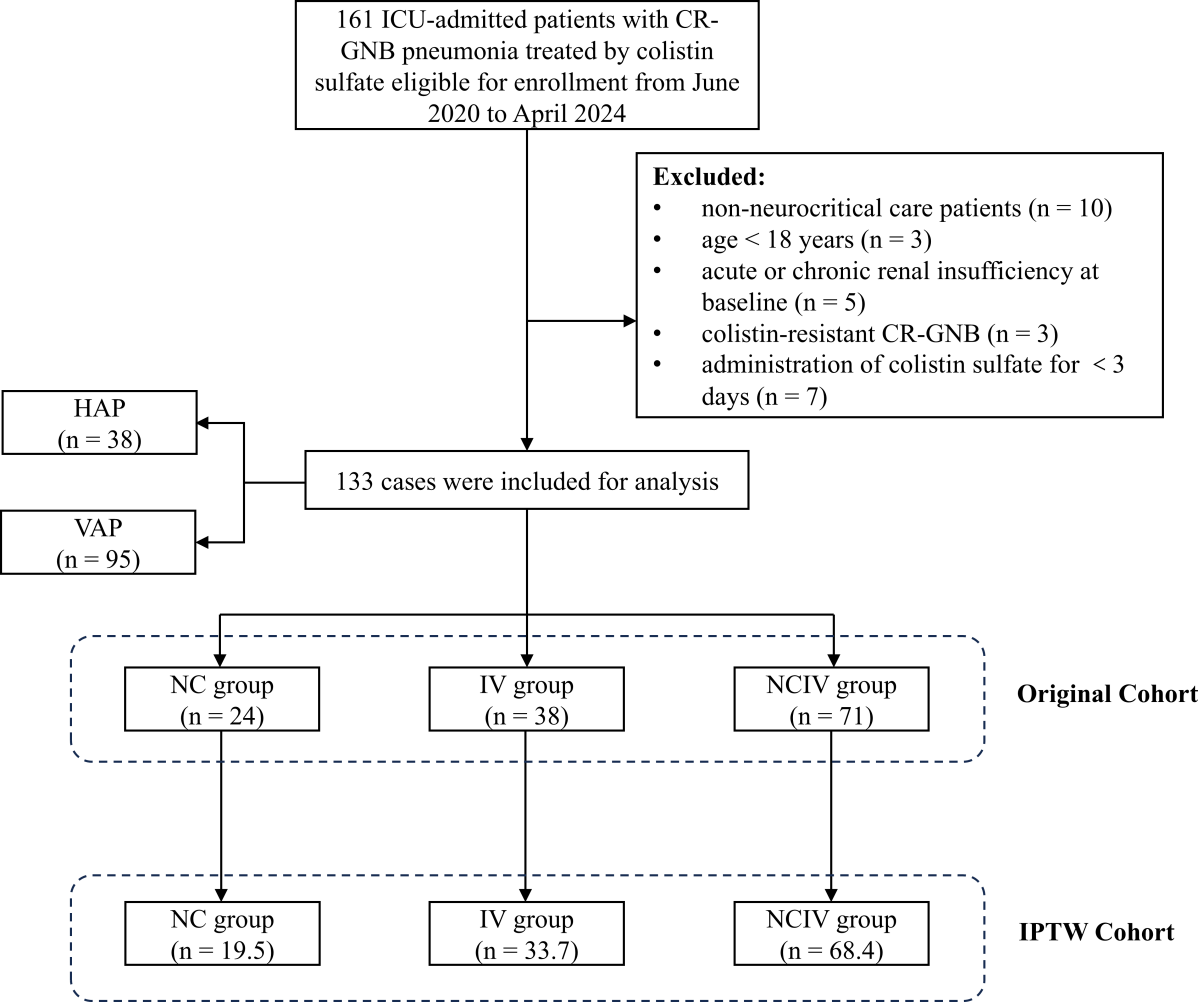
Flowchart of patient recruitment. ICU, intensive care unit; CR-GNB, carbapenem-resistant gram-negative bacteria; HAP, hospital-acquired pneumonia; VAP, ventilator-associated pneumonia; NC, nebulized colistin sulfate alone; IV, intravenous colistin sulfate alone; NCIV, nebulized combination with intravenous colistin sulfate; IPTW, inverse probability of treatment weighting.

A total of 133 neurocritical care patients in the ICU with nosocomial pneumonia caused by CR-GNB were included in this study (Fig. 1). We divided these patients into the NC (*n* = 24), IV (*n* = 38), and NCIV (*n* = 71) groups by different routes of administration of colistin sulfate. Table 1 shows the demographic and clinical features of the study participants and comparisons among the three groups. Patients in the NCIV group were more likely to receive carbapenem treatment than those in the IV group (*P* = 0.002 for pairwise comparison). The GCS score and the PCT level were significantly higher in the IV group than in the NC group (*P* = 0.042 and *P* = 0.032 for pairwise comparisons, respectively). Additionally, the percentage of neutrophils was higher in the IV group than in the other two groups (*P* = 0.008). As shown in Table 1 and Fig. S1, all of the baseline differences were balanced after IPTW, except for residual borderline differences in body mass index (BMI) (SMD = 27.4%), isolated pathogenic bacteria (SMD = 30.1%), tigecycline therapy (SMD = 26.2%), and PCT levels (SMD = 24.7%).

**TABLE 1:** Demographic characteristics and clinical features before and after inverse probability of treatment weighting^*d*^^,^^*e*^

	Original cohort					IPTW cohort				
	NC group (*n* = 24)	IV group (*n* = 38)	NCIV group (*n* = 71)	*P-*value	SMD	NC group (*n* = 19.5)	IV group (*n* = 33.7)	NCIV group (*n* = 68.4)	*P-*value	SMD
Age (years)	50.0 (44.5, 64.0)	57.0 (39.3, 65.5)	59.0 (43.5, 68.0)	0.226	0.299	54.1 (46.0, 64.0)	58.0 (45.6, 64.0)	57.0 (40.8, 67.0)	0.999	0.043
Male, *n* (%)	19 (79.2%)	26 (68.4%)	49 (69.0%)	0.600	0.164	12.9 (65.8%)	23.4 (69.6%)	47.1 (68.9%)	0.969	0.055
BMI (kg/m^2^)	26.1 ± 3.1	23.9 ± 3.8	24.4 ± 4.3	0.083	0.416	26.5 ± 3.3	24.9 ± 4.0	25.1 ± 4.8	0.446	0.274
Smoking history	7 (29.2%)	6 (15.8%)	15 (21.1%)	0.453	0.216	5.3 (26.9%)	10.4 (30.8%)	17.4 (25.5%)	0.911	0.079
Underlying comorbidity										
Chronic pulmonary disease	0 (0.0%)	1 (2.6%)	1 (1.4%)	1	0.163	0.0 (0.0%)	0.4 (1.3%)	0.8 (1.2%)	0.816	0.110
Hypertension	13 (54.2%)	14 (36.8%)	32 (45.1%)	0.403	0.235	7.8 (39.8%)	17.0 (50.4%)	30.4 (44.4%)	0.816	0.143
Diabetes	3 (12.5%)	5 (13.2%)	11 (15.5%)	1	0.058	1.2 (6.1%)	4.8 (14.4%)	9.2 (13.4%)	0.617	0.185
Cardiovascular disease	5 (20.8%)	2 (5.3%)	9 (12.7%)	0.178	0.319	1.8 (9.2%)	3.1 (9.0%)	8.1 (11.8%)	0.877	0.060
Malignancies	5 (20.8%)	4 (10.5%)	11 (15.5%)	0.488	0.191	1.7 (8.8%)	5.1 (15.0%)	9.3 (13.6%)	0.778	0.128
Isolated pathogenic bacteria				0.611	0.336				0.720	0.301
CRAB	19 (79.2%)	25 (65.8%)	48 (67.6%)			14.8 (75.5%)	20.5 (60.9%)	45.4 (66.4%)		
CRE	5 (20.8%)	12 (31.6%)	18 (25.4%)			4.8 (24.5%)	12.5 (37.2%)	19.9 (29.2%)		
CRPA	0 (0.0%)	1 (2.6%)	5 (7.0%)			0.0 (0.0%)	0.6 (1.9%)	3.0 (4.4%)		
Concomitant antibiotic therapy										
Carbapenem	14 (58.3%)	32 (84.2%)	39 (54.9%)^*b*^	**0.008**	0.446	15.2 (77.8%)	22.4 (66.5%)	45.5 (66.5%)	0.675	0.168
Sulbactam	3 (12.5%)	5 (13.2%)	7 (9.9%)	0.811	0.069	4.1 (21.1%)	5.0 (14.7%)	9.2 (13.4%)	0.811	0.137
Tigecycline	2 (8.3%)	7 (18.4%)	20 (28.2%)	0.106	0.354	1.5 (7.6%)	7.1 (21.1%)	14.0 (20.4%)	0.440	0.262
Aminoglycoside	1 (4.2%)	3 (7.9%)	10 (14.1%)	0.410	0.235	3.2 (16.3%)	4.0 (12.0%)	7.0 (9.8%)	0.840	0.129
Ceftazidime-avibactam	4 (16.7%)	4 (10.5%)	12 (16.9%)	0.758	0.124	4.1 (20.9%)	4.3 (12.9%)	9.1 (13.3%)	0.769	0.143
Presenting features^*c*^										
Septic shock	1 (4.2%)	8 (21.1%)	8 (11.3%)	0.152	0.354	2.8 (14.5%)	5.1 (15.1%)	7.8 (11.5%)	0.905	0.071
Invasive ventilator	15 (62.5%)	29 (76.3%)	51 (71.8%)	0.500	0.202	13.9 (71.0%)	21.2 (63.1%)	46.2 (67.5%)	0.888	0.112
GCS score	7.0 (4.8, 9.0)	9.0 (6.3, 10.8)^*a*^	7.0 (5.0, 9.0)	**0.024**	0.416	7.9 (6.0, 9.0)	9.0 (6.0, 10.0)	8.0 (6.0, 10.0)	0.599	0.170
Laboratory results										
Leucocytes (×10^9^/L)	13.2 (9.8, 17.0)	13.8 (10.3, 17.5)	11.4 (8.3, 15.9)	0.384	0.148	12.0 (9.3, 16.8)	13.3 (10.2, 17.3)	11.9 (8.0, 16.6)	0.894	0.105
Neutrophil (%)	78.5 ± 9.7	85.6 ± 8.8^*a*^	81.1 ± 9.3^*b*^	**0.008**	0.512	83.2 ± 8.6	83.7 ± 8.9	81.8 ± 9.1	0.641	0.137
CRP (mg/L)	55.0 (15.8, 85.2)	67.9 (23.7, 153.9)	71.8 (30.5, 113.8)	0.226	0.368	79.7 (23.4, 110.5)	57.7 (17.6, 78.6)	66.1 (19.6, 109.5)	0.851	0.052
PCT (ng/mL)	0.1 (0.1, 0.2)	0.3 (0.1, 2.1)^*a*^	0.2 (0.1, 0.9)	**0.038**	0.420	0.1 (0.1, 0.2)	0.2 (0.1, 0.9)	0.2 (0.1, 0.8)	0.709	0.247
Albumin (g/L)	32.8 ± 3.5	31.9 ± 5.4	32.4 ± 4.4	0.710	0.143	32.7 ± 2.9	32.1 ± 5.5	32.9 ± 4.8	0.870	0.104
Serum creatinine (μmol/L)	58.0 (44.9, 84.3)	49.4 (41.3, 66.4)	53.8 (41.4, 68.8)	0.544	0.261	56.3 (46.3, 69.5)	50.4 (41.4, 65.4)	54.4 (44.1, 69.2)	0.842	0.113
GFR (mL/min)	138.0 (101.1, 186.1)	129.3 (105.1, 172.9)	124.2 (96.1, 163.4)	0.664	0.102	129.4 (82.9, 182.6)	123.6 (102.1, 156.4)	124.3 (95.3, 167.2)	0.922	0.085
ARC, *n* (%)	13 (54.2%)	19 (50.0%)	34 (47.9%)	0.867	0.084	10.2 (52.2%)	16.0 (47.6%)	33.6 (49.2%)	0.966	0.061

^*a*^ *P* < 0.05 when compared with the NC group.

^*b*^ *P* < 0.05 when compared with the IV group.

^*c*^ Presence of organ dysfunction on the pneumonia index date.

^*d*^ Data were presented as mean ± standard deviation (SD), median (interquartile ranges, IQR), or number (percentage). Boldface denotes statistically significant values (*P* < 0.05).

^*e*^ IPTW, inverse probability of treatment weighting; NC, nebulized colistin sulfate alone; IV, intravenous colistin sulfate alone; NCIV, nebulized combination with intravenous colistin sulfate; SMD, standardized mean difference; BMI, body mass index; CRAB, carbapenem-resistant *Acinetobacter baumannii*; CRE, carbapenem-resistant* Enterobacteriaceae*; CRPA, carbapenem-resistant* Pseudomonas aeruginosa*; GCS, Glasgow Coma Scale; CRP, C-reactive protein; PCT, procalcitonin; GFR, glomerular filtration rate; ARC, augmented renal clearance.

### Efficacy outcomes

In the original cohort, patients in the IV group had higher clinical failure rates on days 7 and 14 than the NC (36.8% vs. 20.8%, *P* = 0.028 and 26.3% vs. 8.3%, *P* = 0.035, respectively) and NCIV (36.8% vs. 23.9%, *P* = 0.018 and 26.3% vs. 14.1%, *P* = 0.020, respectively) groups but had comparable outcomes on day 28 (Table S1). There were significant differences in the microbiological eradication rate on day 7 between the NC and IV groups (62.5% vs. 34.4%, *P* = 0.038), and NCIV demonstrated a higher microbiological eradication rate than IV on day 14 (85.0% vs. 65.6%, *P* = 0.019) (Table S1). After IPTW, the clinical failure rates on days 7 and 14 were still significantly higher in the IV group when compared with the NC group (38.3% vs. 20.5%, *P* = 0.017 and 32.1% vs. 15.3%, *P* = 0.004, respectively) and the NCIV group (38.3% vs. 24.7%, *P* = 0.023 and 32.1% vs. 14.2%, *P* = 0.015, respectively) (Table 2). Furthermore, the IV group reported a lower microbiological eradication rate on day 14 (69.4% vs. 86.2%, *P* = 0.031), as well as longer ICU (*P* = 0.020) and hospital stays (*P* = 0.037) than the NCIV group. Notably, the IV group also exhibited longer ICU and hospital stays than the NC group (*P* = 0.039and *P* = 0.018, respectively) (Table 2).

**TABLE 2:** Treatment outcomes of neurocritical care patients treated with colistin sulfate after inverse probability of treatment weighting^*c*^^,^^*d*^

	IPTW cohort					
	NC group (*n* = 19.5)	IV group (*n* = 33.7)	NCIV group (*n* = 68.4)	*p*_1_*-*value	*p*_2_*-*value	*p*_3_*-*value
Clinical failure						
Day 7	4.0 (20.5%)	12.9 (38.3%)	16.9 (24.7%)	**0.017**	0.270	**0.023**
Day 14	3.0 (15.3%)	10.8 (32.1%)	9.7 (14.2%)	**0.004**	0.308	**0.015**
Day 28	3.0 (15.3%)	2.9 (8.5%)	4.8 (7.0%)	0.573	0.344	0.823
Microbiological eradication^*a*^						
Day 7	12.2/19.5 (62.2%)	11.4/27.0 (42.2%)	30.5/61.3 (49.8%)	0.151	0.544	0.187
Day 14	17.1/18.8 (91.3%)	18.8/27.0 (69.4%)	48.2/55.9 (86.2%)	0.051	0.967	**0.031**
Day 28	17.7/18.8 (94.4%)	24.5/26.6 (92.1%)	50.2/54.8 (91.7%)	0.331	0.322	0.660
All-cause mortality						
Day 14	0.5 (2.8%)	0.7 (2.1%)	6.9 (10.1%)	0.984	0.111	0.071
Day 28	1.0 (4.9%)	2.1 (6.3%)	9.3 (13.6%)	0.865	0.662	0.478
28-Day ventilator weaning^*b*^						
Day 28	12.4/13.9 (89.1%)	14.2/21.2 (67.0%)	27.9/46.2 (60.5%)	0.556	0.416	0.739
ICU stays (median, IQR)	23.3 (17.7, 30.0)	30.0 (24.0, 59.8)	23.0 (17.1, 39.0)	**0.039**	0.681	**0.020**
Hospital stays (median, IQR)	27.4 (19.8, 32.0)	35.0 (26.5, 72.1)	32.0 (22.0, 48.0)	**0.018**	0.706	**0.037**
Nephrotoxicity (KDIGO criteria)	1.2 (6.1%)	3.1 (9.4%)	8.5 (12.4%)	0.367	0.506	0.576
Stage 1	0.8 (4.3%)	2.7 (8.1%)	2.8 (4.1%)			
Stage 2	0 (0.0%)	0 (0.0%)	2.5 (3.6%)			
Stage 3	0.4 (1.8%)	0.4 (1.3%)	3.2 (4.7%)			

^*a*^ Patients were excluded from the analysis because sputum culture was not adequately performed.

^*b*^ Only cases with invasive ventilators were included for analysis.

^*c*^ Boldface denotes statistically significant values (*P* < 0.05).

^*d*^ IPTW, inverse probability of treatment weighting; NC, nebulized colistin sulfate alone; IV, intravenous colistin sulfate alone; NCIV, nebulized combination with intravenous colistin sulfate; ICU, intensive care unit; IQR, interquartile range; KDIGO, Kidney Disease: Improving Global Outcomes. *p*_1_ represents the comparison between the NC and IV groups; *p*_2_ represents the comparison between the NC and NCIV groups; and *p*_3_ represents the comparison between the IV and NCIV groups.

**Fig 2:**
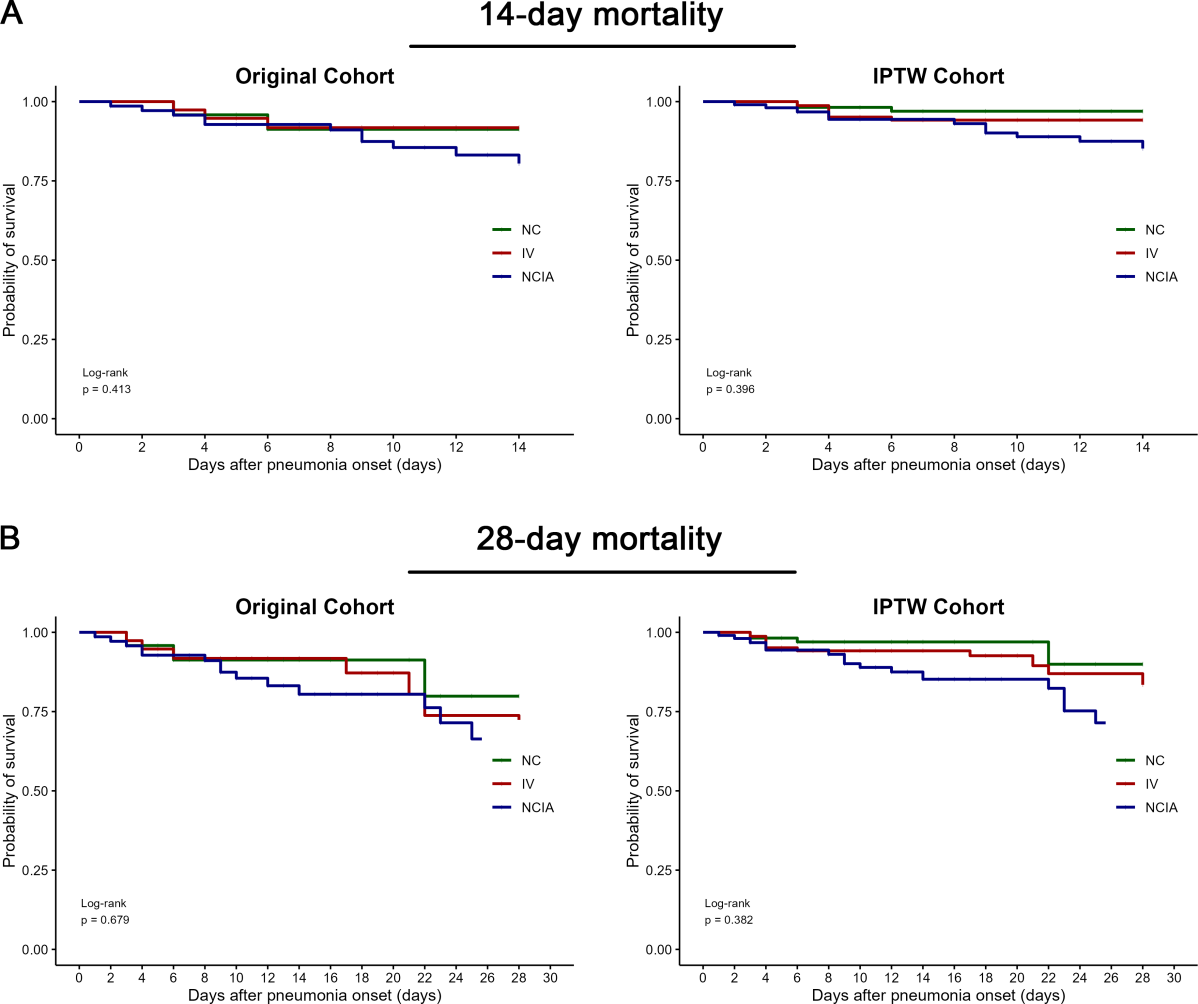
Kaplan-Meier analyses of (**A**) 14- and (**B**) 28-day mortality among the three groups. IPTW, inverse probability of treatment weighting; NC, nebulized colistin sulfate alone; IV, intravenous colistin sulfate alone; NCIV, nebulized combination with intravenous colistin sulfate.

The results of the Kaplan-Meier analyses of all-cause mortalities in the original and IPTW cohorts are shown in Fig. 2. There was no significant difference in the 14- or 28-day mortality risk among the three groups in both cohorts.

### Safety outcome

Nephrotoxicity was assessed using the KDIGO criteria after the initiation of colistin sulfate therapy. A total of 12.5, 10.5, and 16.9% of patients in the NC, IV, and NCIV groups, respectively, developed acute kidney injury (AKI), but we did not observe significant differences among these three groups (Table S1). After IPTW, the occurrence of AKI was comparable among the three groups (Table 2). In this study, none of the patients required renal replacement therapy.

### Independent factors associated with clinical failure

Univariable and multivariable analyses of clinical factors associated with clinical failure on day 14 were performed on the original and IPTW cohorts (Table 3). In the multivariable analysis, combination therapy with sulbactam (adjusted odds ratio [aOR] = 4.58, 95% confidence interval [CI] = 1.35–15.60, *P* = 0.032) and high CRP (aOR = 1.01, 95% CI = 1.00–1.02, *P* = 0.012) and GFR (aOR = 1.01, 95% CI = 1.00–1.02, *P* = 0.011) levels were significantly associated with greater clinical failure in the original cohort. In the IPTW cohort, the independent factors associated with clinical failure on day 14 included combination therapy with sulbactam (aOR = 4.62, 95% CI = 1.16–18.43, *P* = 0.030), GFR (aOR = 1.01, 95% CI = 1.00–1.02, *P* = 0.003), and intravenous colistin sulfate alone (aOR = 5.92, 95% CI = 1.14–30.84, *P* = 0.035).

**TABLE 3:** Univariable and multivariable analyses of clinical factors associated with clinical failure on day 14^*a*^^,^^*b*^

	Original cohort				IPTW cohort			
	Univariable analysis		Multivariable analysis		Univariable analysis		Multivariable analysis	
	OR (95% CI)	*P-*value	aOR (95% CI)	*P-*value	OR (95% CI)	*P-*value	aOR (95% CI)	*P-*value
Age	1.04 (0.99–1.10)	0.077	1.02 (0.98–1.07)	0.199	1.04 (0.96–1.14)	0.310		
Male (reference)	0.62 (0.10–3.06)	0.575			0.63 (0.13–2.96)	0.551		
BMI	0.94 (0.78–1.13)	0.507			0.95 (0.79–1.13)	0.558		
Smoking history	1.80 (0.32–9.02)	0.479			3.84 (0.53–27.75)	0.180		
Underlying comorbidity								
Chronic pulmonary disease	0.00 (0.00–0.00)	0.992			0.00 (0.00–0.00)	0.994		
Hypertension	0.72 (0.15–3.44)	0.683			0.59 (0.14–2.56)	0.481		
Diabetes	0.21 (0.01–1.82)	0.200			0.06 (0.003–0.98)	**0.049**	0.09 (0.01–1.39)	0.084
Cardiovascular disease	1.80 (0.15–17.18)	0.614			1.58 (0.15–16.40)	0.700		
Malignancies	8.80 (1.36–64.46)	**0.024**	2.51 (0.65–9.69)	0.207	3.67 (0.61–22.00)	0.153		
Isolated pathogenic bacteria								
CRAB (reference)								
CRE	0.74 (0.14–3.28)	0.695			0.51 (0.05–5.38)	0.576		
CRPA	1.27 (0.05–15.28)	0.862			1.69 (0.11–27.16)	0.708		
Intravenous antibiotics								
Carbapenem	0.47 (0.09–2.30)	0.343			0.66 (0.12–3.68)	0.633		
Sulbactam	9.62 (1.50–73.65)	**0.020**	4.58 (1.35–15.60)	**0.032**	11.96 (2.45–61.13)	**0.003**	4.62 (1.16–18.43)	**0.030**
Tigecycline	3.69 (0.73–19.86)	0.114			4.80 (0.56–41.16)	0.151		
Aminoglycoside	0.26 (0.02–2.07)	0.240			0.12 (0.01–2.71)	0.178		
Ceftazidime-avibactam	5.42 (0.84–37.83)	0.075	3.43 (0.87–13.53)	0.095	7.50 (1.03–54.77)	0.047	2.10 (0.52–8.51)	0.297
Presenting features								
Septic shock	1.66 (0.20–11.63)	0.618			1.27 (0.26–6.09)	0.765		
Invasive ventilator	0.46 (0.10–2.14)	0.314			0.26 (0.05–1.49)	0.129		
GCS score	0.83 (0.57–1.19)	0.321			0.93 (0.61–1.43)	0.749		
Laboratory results								
Leucocytes	0.97 (0.87–1.08)	0.598			1.00 (0.88–1.14)	0.956		
Neutrophil	1.02 (0.95–1.12)	0.576			1.03 (0.94–1.12)	0.537		
CRP	1.01 (1.00–1.02)	**0.040**	1.01 (1.00–1.02)	**0.012**	1.01 (1.00–1.03)	0.081	1.00 (1.00–1.01)	0.328
PCT	1.00 (0.95–1.06)	0.897			1.01 (0.94–1.10)	0.744		
Albumin	1.06 (0.90–1.26)	0.486			1.09 (0.88–1.34)	0.414		
Serum creatinine	1.01 (0.96–1.03)	0.586			1.01 (0.99–1.04)	0.227		
GFR	1.02 (1.00–1.04)	**0.036**	1.01 (1.00–1.02)	**0.011**	1.02 (1.00–1.04)	**0.032**	1.01 (1.00–1.02)	**0.003**
ARC	2.90 (0.28–32.26)	0.370			4.72 (0.42–53.13)	0.206		
Route of administration								
Nebulized colistin sulfate alone (reference)								
Intravenous colistin sulfate alone	12.68 (1.43–175.48)	**0.035**	3.86 (0.73–20.42)	0.130	15.36 (2.41–98.08)	**0.004**	5.92 (1.14–30.84)	**0.035**
Nebulized and intravenous colistin sulfate	1.74 (0.23–18.66)	0.611	1.49 (0.33–6.64)	0.651	2.97 (0.36–24.50)	0.308	1.76 (0.33–9.45)	0.510

^*a*^ IPTW, inverse probability of treatment weighting; OR, odds ratio; CI, confidence interval; aOR, adjusted OR; BMI, body mass index; CRAB, carbapenem-resistant *Acinetobacter baumannii*; CRE, carbapenem-resistant* Enterobacteriaceae*; CRPA, carbapenem-resistant* Pseudomonas aeruginosa*; GCS, Glasgow Coma Scale; CRP, C-reactive protein; PCT, procalcitonin; GFR, glomerular filtration rate; ARC, augmented renal clearance.

^*b*^ Boldface denotes statistically significant values (*P* < 0.05).

## DISCUSSION

Respiratory infection is the most common infection in ICU patients. Neurocritical care patients, especially elderly patients, are liable to have pneumonia due to the weakened defense function of the respiratory tract and immunosuppression after brain injury. The incidence of clinical VAP was 12–20% in neurologically injured patients (23). Teng et al. (14) reported that neurosurgical ICU patients with no history of antibiotic use before admission had a 15.7% incidence of MDR bacteria during 7 days of mechanical ventilation. Over the last two decades, polymyxins have shown promise due to the emergence of extensively drug-resistant GNB. PMB and colistin are currently considered last-line defenses against GNB, and they have been used with variable success in several regions worldwide (24). CMS is an inactive prodrug of colistin that is hydrolyzed to multiple components with bactericidal activity (25), while colistin sulfate is administered in its active form. Polymyxins rapidly kill bacteria through direct interaction with the lipid A component of the lipopolysaccharide in the GNB cell wall (26). Mounting evidence has depicted the pharmacokinetics (PK) and pharmacodynamics (PD) of nebulized and intravenous CMS (27–29). Given that the spontaneous conversion to colistin is relatively slow compared with the renal clearance of CMS, only a small proportion of CMS is converted to colistin (25). Consequently, the concentration of active colistin cannot create optimal conditions to treat HAP and VAP efficiently. A hotly debated issue is whether nebulized administration increases the effectiveness of CMS for treating respiratory tract infections caused by CR-GNB.

PMB was topically administered for prophylaxis of GNB pneumonia from the early 1970s (30). Aerosol PMB could effectively prevent bronchial GNB airway colonization, but it was subsequently abandoned due to potential risks. Ten years later, nebulized colistin was advanced as a treatment for patients with cystic fibrosis (7), and its effect on airway clearance of GNB in non-cystic fibrosis bronchiectasis (31), severe chronic obstructive pulmonary disease (COPD) (32), VAP, and tracheobronchitis (25) was further confirmed. Colistin sulfate for parenteral use is available only in China, whose PK, clinical efficacy, and side effects have not been clearly reported. The prescribing information for colistin sulfate recommends an intravenous dosing regimen of 1 to 1.5 million IU per day, divided into two to three administrations. While colistin sulfate and PMB share similar chemical structures and metabolic pathways, PMB is supported by stronger clinical evidence and more established treatment guidelines. The PK profiles of these two agents are remarkably similar, as demonstrated by their comparable PK parameters (33). Later, Xie et al. (34) corroborated these findings and further indicated that the current dosing regimen might be inadequate for patients with CrCl ≥ 80 mL/min or for infections involving pathogens with MIC ≥ 1.0 mg/L. Nevertheless, due to the limited sample size, additional studies and external validation are required to verify the conclusions. Moreover, there is currently a lack of PK/PD data on aerosolized colistin sulfate. In China, a multi-disciplinary expert consensus recommended an inhaled dose of colistin sulfate at 0.25–0.5 million IU twice daily (35). Bao et al. (36) found that adjunctive nebulized colistin sulfate in combination with intravenous antibiotics significantly improved the clinical efficacy in MDR-GNB pneumonia. Our study also emphasized the role of adjunctive nebulized colistin sulfate in favorable clinical outcomes on days 7 and 14, and nebulized colistin sulfate alone could achieve pretty much the same results. In the multivariable analysis of the IPTW cohort, we also identified intravenous colistin sulfate alone as an independent factor associated with greater clinical failure on day 14.

With respect to the microbiological eradication, our findings aligned with previous research (35), which reported a lower bacterial detection rate in the NCIV group than the IV group on day 14. It is noteworthy that only six patients in our cohort had polymicrobial infection or coinfection, so its eventual role in treatment outcomes was not further analyzed.

In 2021, a meta-analysis revealed improved clinical and mortality outcomes in patients with MDR pneumonia receiving nebulized CMS alone or adjunctive CMS compared to those receiving intravenous CMS alone (37). Several studies have shown that nebulized CMS does not improve all-cause mortality, which may be explained by inappropriate dosing or initial empirical antimicrobial therapy (8, 11). The findings from the Cox regression analysis demonstrated no difference in the risk of death among the three groups, but the NCIV group tended to have a higher all-cause mortality. Due to concerns about the varying severity of pneumonia, we speculated that adjunctive colistin sulfate was more likely to be used as salvage therapy for patients with severe infection.

Previous studies have explored the role of nebulized CMS in shortening the length of mechanical ventilation (MV) and ICU stay. Zhang et al. (38) concluded that nebulized CMS was not significantly different in days of MV and ICU stay from intravenous CMS. Moreover, aerosolized plus intravenous CMS also did not affect the length of ICU stay (39). However, our study found that the NC and NCIV groups had shorter ICU and hospital stays than the IV group. Another point worth emphasizing is the potential nephrotoxicity of polymyxins. Nebulized CMS showed significantly lower nephrotoxicity than intravenous CMS (9, 40), and the safety of adjunctive CMS has been confirmed in numerous studies (11, 37). Our results demonstrated no significant difference in the rate of nephrotoxicity among the three groups. Other adverse reactions of polymyxins, such as airway hyperresponsiveness and skin pigmentation, were not observed in our cohort.

To our knowledge, this is the first study evaluating the efficacy and safety of colistin sulfate in neurocritical care patients. While retrospective studies are generally prone to immortal time bias, our uniform time-zero definition and strict inclusion criteria substantially mitigated this risk. In this study, only patients who received colistin sulfate within 5 days before or after the index culture study were included, with survival analysis uniformly calculated from the first day of colistin sulfate administration in all groups. To interpret the results more appropriately, four main limitations need to be considered. First, this study was designed as a retrospective cohort study, which may lead to bias due to uncontrolled confounding variables. Notably, the treatment regimens of colistin sulfate were not randomized but partly based on the severity of symptoms. IPTW was employed to create a pseudo-population in which measured confounders were equally distributed across groups to minimize the impact of treatment selection bias and other potential confounders. Second, the sample size of our study was relatively small, which limited the statistical power to detect potentially smaller magnitude associations. The sample size of the NC group (*n* = 24) was especially small and may have impacted the reliability of the comparisons. Third, the retrospective nature of our study led to the limitation of clinical data, such as the Acute Physiology and Chronic Health Evaluation (APACHE) II score and Sequential Organ Failure Assessment (SOFA) score, which may have amplified the bias. Finally, no standardized protocol for nebulized colistin sulfate, including dosage, duration, and types of nebulizer systems, had been developed in our study. For future analyses, it is recommended to implement lung tissue concentration measurements combined with therapeutic drug monitoring (TDM) to facilitate dose optimization. A high dose of nebulized CMS is actually recommended for pneumonia (25, 41), and further studies are warranted to confirm these findings.

## CONCLUSIONS

This study retrospectively collected data about neurocritical care patients in the ICU with nosocomial pneumonia caused by CR-GNB, who were treated with different treatment regimens of colistin sulfate. Our study suggested that nebulized colistin sulfate with concurrent intravenous administration may be an effective and safe treatment option for nosocomial infections caused by CR-GNB. To further explore the best available therapy of nebulized colistin sulfate, larger sample sizes and prospective studies are needed to elucidate its clinical benefits.

## SUPPLEMENTAL MATERIAL

Supplemental material aac.00644-25-s0001.docx Fig. S1; Table S1.

ASM does not own the copyrights to Supplemental Material that may be linked to, or accessed through, an article. The authors have granted ASM a non-exclusive, world-wide license to publish the Supplemental Material files. Please contact the corresponding author directly for reuse.
