# Supplementary material for: Comparison of different colistin sulfate regimens for carbapenem-resistant gram-negative bacteria pneumonia in neurocritical care patients: a retrospective cohort study
Source: Antimicrob Agents Chemother. 2025 Sep 22;69(11):e00644-25. doi: 10.1128/aac.00644-25 (PMC12617292; doi:10.1128/aac.00644-25)
Supplement: Supplemental material — Fig. S1; Table S1. [file aac.00644-25-s0001.docx]

**Fig. S1** Standardized mean difference (SMD) of baseline variables before and after inverse probability of treatment weighting (IPTW). PCT, procalcitonin; GCS, Glasgow Coma Scale; BMI, body mass index; CRP, C-reactive protein; GFR, glomerular filtration rate; ARC, augmented renal clearance.


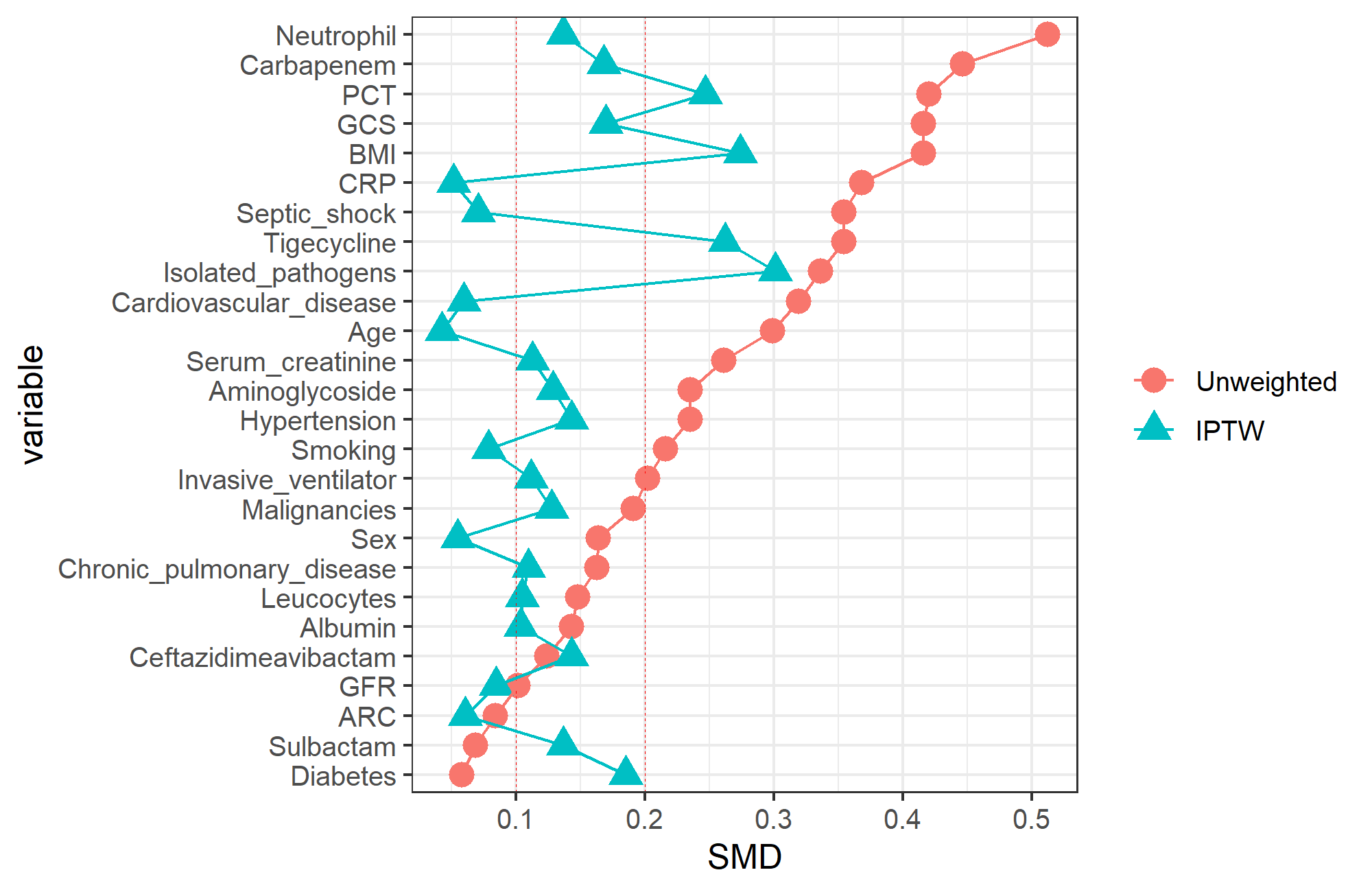


**Table S1**. Treatment outcomes of neurocritical care patients treated with colistin sulfate before inverse probability of treatment weighting

|  | Original Cohort | | | | | |
| --- | --- | --- | --- | --- | --- | --- |
|  | NC group (n = 24) | IV group (n = 38) | NCIV group (n = 71) | *p*_1_*-*value | *p*_2_*-*value | *p*_3_*-*value |
| Clinical failure | | | | | | |
| Day 7 | 5 (20.8%) | 14 (36.8%) | 17 (23.9%) | **0.028** | 0.561 | **0.018** |
| Day 14 | 2 (8.3%) | 10 (26.3%) | 10 (14.1%) | **0.035** | 0.611 | **0.020** |
| Day 28 | 2 (8.3%) | 5 (13.2%) | 7 (9.9%) | 0.374 | 0.324 | 0.938 |
| Microbiological eradication^a^ | | | | | | |
| Day 7 | 15/24 (62.5%) | 11/32 (34.4%) | 32/65 (49.2%) | **0.038** | 0.258 | 0.118 |
| Day 14 | 20/23 (87.0%) | 21/32 (65.6%) | 51/60 (85.0%) | 0.057 | 0.827 | **0.019** |
| Day 28 | 22/23 (95.7%) | 27/31 (87.1%) | 53/59 (89.8%) | 0.244 | 0.505 | 0.340 |
| All-cause mortality | | | | | | |
| Day 14 | 2 (8.3%) | 2 (5.3%) | 10 (14.1%) | 0.884 | 0.250 | 0.135 |
| Day 28 | 3 (12.5%) | 6 (15.8%) | 13 (18.3%) | 0.691 | 0.999 | 0.550 |
| 28-day ventilator weaning^b^ | | | | | | |
| Day 28 | 12/15 (80.0%) | 19/29 (65.5%) | 28/51 (54.9%) | 0.503 | 0.094 | 0.364 |
| ICU stays (Median, IQR) | 28.0 (18.0, 32.5) | 30.0 (24.0, 51.5) | 23.0 (18.0, 41.0) | 0.124 | 0.785 | 0.073 |
| Hospital stays (Median, IQR) | 30.0 (22.5, 34.0) | 36.0 (26.3, 62.3) | 34.0 (24.0, 55.0) | 0.067 | 0.486 | 0.088 |
| Nephrotoxicity (KDIGO criteria) | 3 (12.5%) | 4 (10.5%) | 12 (16.9%) | 0.154 | 0.291 | 0.438 |
| Stage 1 | 2 (8.3%) | 3 (7.9%) | 4 (5.6%) |  |  |  |
| Stage 2 | 0 (0.0%) | 0 (0.0%) | 3 (4.2%) |  |  |  |
| Stage 3 | 1 (4.2%) | 1 (2.6%) | 5 (7.1%) |  |  |  |

NC, nebulized colistin sulfate alone; IV, intravenous colistin sulfate alone; NCIV, nebulized combination with intravenous colistin sulfate; ICU, intensive care unit; IQR, interquartile range; KDIGO, Kidney Disease: Improving Global Outcomes.

*p*_1_ represented the comparison between the NC group and the IV group; *p*_2_ represented the comparison between the NC group and the NCIV group; *p*_3_ represented the comparison between the IV group and the NCIV group.

^a^ Patients were excluded from the analysis because sputum culture was not adequately performed.

^b^ Only cases with invasive ventilators were included for analysis.

Boldface denotes statistically significant values (*p* < 0.05).
